# Impact of COVID-19 Pandemic on Clinical Care of Peripheral Arterial Disease Patients: A Single-Center Experience

**DOI:** 10.3390/jcm12030890

**Published:** 2023-01-23

**Authors:** Elias Noory, Tanja Böhme, Jonas Salm, Ulrich Beschorner, Dirk Westermann, Thomas Zeller

**Affiliations:** Clinic for Cardiology and Angiology, University Heart Center Freiburg-Bad Krozingen, Campus Bad Krozingen, Südring 15, 79189 Bad Krozingen, Germany

**Keywords:** COVID-19 pandemic, peripheral artery disease, amputation

## Abstract

**Highlights:**

**What are the main findings?**

- This study not only focuses on the first wave of the pandemic, but also on its subsequent progression.

**What is the implication of the main finding?**

- This study shows an increase in peripheral interventions of patients referred with wounds requiring more complex interventions. However, as a potential benefit of the endovascular treatment in a high volume center with mainly experienced operators, no increase in overall acute amputations was found.

**Abstract:**

Objective: To better manage the burden of the COVID-19 pandemic on hospitals, numerous scheduled procedures have been postponed nationwide. Design and Methods: Retrospective analysis of patient characteristics and outcomes of patients hospitalized with peripheral arterial disease (PAD) in the period prior to the COVID-19 pandemic (2018 and 2019) and during the pandemic (2020 and 2021). This study assesses the in-hospital outcomes. Main endpoints are Rutherford stages at admission for intervention, incidence of amputation, of total occlusion, and duration of intervention. The data were analyzed descriptively. Results: The total number of interventions due to PAD had decreased in 2020, but not significantly during the pandemic period (*n* = 5351) compared to the period prior to COVID-19 pandemic (*n* = 5351) (*p* = 0.589). The proportion of interventions treated for critical limb-threatening ischaemia (CLTI) increased from 2018/2019 (*n* = 2112) to 2020/2021 (*n* = 2426) (*p* < 0.001). However, the proportion of patients with wounds requiring amputation was not higher during the pandemic (*n* = 191) than before (*n* = 176) (minor amputations *p* = 0.2302, major amputations *p* = 0.9803). The proportion of total occlusions did not differ significantly between the pre-COVID-19 (*n* = 3082) and the COVID-19 pandemic periods (*n* = 2996) (*p* = 0.8207). Multilevel interventions did not increase significantly from 2018/2019 (*n* = 1930) to 2020/2021 (*n* = 2071). Between 2018/2019 and 2020/2021, the procedure duration and fluoroscopy duration increased significantly. However, parameters such as contrast agent volume and radiation dose did not differ significantly. The average length of stay was 4.6 days. Conclusion: The COVID-19 pandemic had an impact on the in-patient care of PAD patients in terms of disease stage severity and complexity. However, the amputation rate was not affected.

## 1. Introduction

The COVID-19 pandemic has posed major challenges to societies and especially to the health systems. Elective interventions and surgical procedures were reduced or completely suspended in order to bundle resources for COVID-19 patient care.

The number of emergency department consultations declined during the pandemic [1,2].

Globally, a decrease in admissions of patients with acute myocardial infarction was also reported during the pandemic [3,4,5].

To date, there is limited evidence on the impact on patients with peripheral arterial disease (PAD) during the COVID-19 pandemic.

An Australian/New Zealand study showed a significant decrease in vascular surgery procedures in 2020 compared to the period of January–September 2015–2019. The decrease resulted from the reduced number of elective procedures, while the number of emergency procedures increased during this period [6].

A Swiss study showed that fewer patients underwent vascular surgery in 2020 than in 2018, but the proportion of patients with acute critical limb ischemia was higher [7]. Another study showed a decrease in deferred surgical procedures during the COVID-19 pandemic [8].

The aim of this retrospective study was to investigate the impact of the COVID-19 pandemic on the clinical care of patients with PAD in a German high-volume interventional center, focusing on the disease complexity and the patient’s post-procedural clinical outcome.

## 2. Methods

An internal prospective database of the department was retrospectively analyzed. All endovascular interventions for PAD from January 2018 until December 2021 were included and the in-hospital outcome was assessed. There were no inclusion and exclusion criteria.

The Rutherford–Becker stage on admission, vessel segments treated (separated into iliac, femoropopliteal, and tibial vessels), presence of total occlusion, treatment duration, volume of contrast medium, and radiation dose were documented and evaluated.

In addition, the amputations performed during the hospital stay were queried. A distinction was made between major (defined as proximal to the tarsometatarsal joint) and minor (defined as toe or transmetatarsal amputations) amputations. For patients with wounds, discharge recommendations were analyzed to identify transfers to other hospitals for amputation.

The study was approved by the institutional review board.

### 2.1. Study Endpoints

The primary study endpoint is the incidence of critical limb-threatening ischemia (CLTI) on hospital admission prior to and during the COVID-19 pandemic. Chronic limb-threatening ischemia (CLTI) is a clinical syndrome defined by the presence of PAD in combination with rest pain, gangrene, or a lower limb ulceration of more than two weeks’ duration and is associated with increased risk of mortality and amputation, and impaired quality of life [9].

Secondary endpoints included the number of amputations and the number of transfers to another hospital for amputation. In addition, the complexity of the interventions is evaluated based on the proportion of recanalizations of total occlusions, the treated vessel segments, the duration of the intervention, the volume of contrast medium, and the radiation dose. For this purpose, the interventional procedures of three operators were evaluated regarding the intervention-related endpoints and compared between the two study periods.

### 2.2. Statistical Analysis

All analyses were performed using SPSS software (version 23.0; SPSS, Chicago, IL, USA), the free software for statistical computing and graphics R (R 4.2.0; R Foundation, Vienna, Austria) and EXCEL (Windows X, Microsoft, Redmond, Washington, DC, USA). Categorical variables are expressed as frequencies and percentages. Continuous variables are expressed as mean values with standard deviations. Differences between groups were tested using the chi-squared test for categorical variables, and the Student’s *t*-test for continuous variables. All tests were two-tailed. The significance level was set to α  =  0.05.

## 3. Results

### 3.1. Number of Peripheral Interventions before (2018/2019) and during the COVID-19 Pandemic (2020/2021)

While there were 5361 endovascular interventions due to PAD in 2018 and 2019, there were 5190 interventions during the COVID-19 pandemic years 2020 and 2021 (*p* = 0.589). The reduction in interventions was mainly due to fewer interventions in 2020 (2020 *n* = 2522, 2021 *n* = 2668). Only 17 patients tested positive for COVID-19 during hospitalization. Patients’ cardiovascular risk factors are shown in Table 1 with no significant differences between the two cohorts. The proportion of interventions performed for CLTI increased from 2018/2019 to 2020/2021 (*p* < 0.001, see Table 2). In 2018 and 2019, the length of hospital stay was 4.7 ± 4.5 days and in 2020 and 2021 4.6 ± 5.5 days (*p* = 0.549).

**Figure 1:**
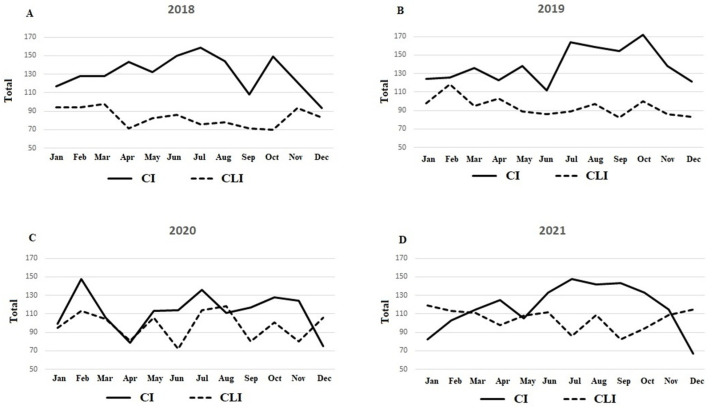
(**A**–**D**) Title: Number of interventions performed for intermittent claudication and CLTI for 2018 to 2021 by month.Legend: Number of interventions performed for intermittent claudication and CLTI for 2018 to 2021 by month. IC—intermittent claudication, CLTI critical limb-threatening ischaemia.

Figure 1 shows the number of interventions performed for intermittent claudication and CLTI for 2018 to 2021 by month.

### 3.2. Procedural Characteristics and Outcomes

In 2018 and 2019, the proportion of recanalizations was 57.5% (*n* = 3082). During the COVID-19 pandemic years of 2020 and 2021, there were 2996 recanalizations (57.7%) (*p* = 0.820).

While the number of iliac interventions remained stable at 572, 583 and 584 in 2018 2019, and 2021, respectively, there was a decrease to 502 iliac interventions in 2020. Regarding femoropopliteal interventions, 4190 procedures were performed in 2018/2019. During the COVID-19 pandemic (2020/2021), the number was 4085 (*p* = 0.690). The proportion of femoropopliteal interventions was stable in both study periods at 78.2% and 78.7%, respectively. The proportion of infrapopliteal interventions increased from 37.7% in 2018/2019 to 41.9% during the COVID-19 pandemic years 2020/2021 (*p* = 0.100). Multilevel interventions increased from 2018 to 2021. None of these changes were significantly different during the COVID-19 pandemic compared to the pre-COVID-19 period. Details are shown in Table 3.

The peri-procedural details are given in Table 4. Between 2018/2019 and 2020/2021, the procedure duration and fluoroscopy duration increased significantly. The contrast volume, and radiation dose, however, did not differ significantly.

### 3.3. Acute Limb Loss Outcomes

**Figure 2:**
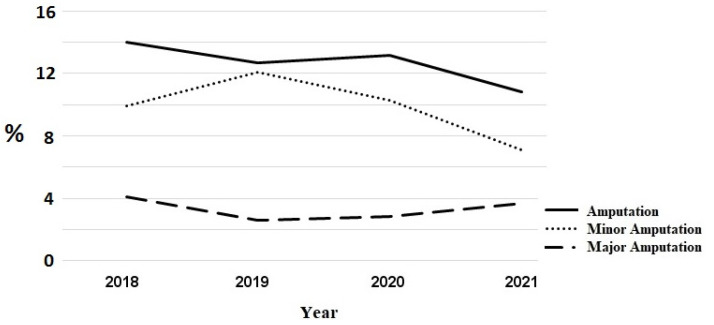
Title: Proportionate course of in-house amputations in patients with Rutherford–Becker class 5 (including minor and major). Legend: Proportionate major and minor in-house amputations in patients with Rutherford–Becker class 5.

**Figure 3:**
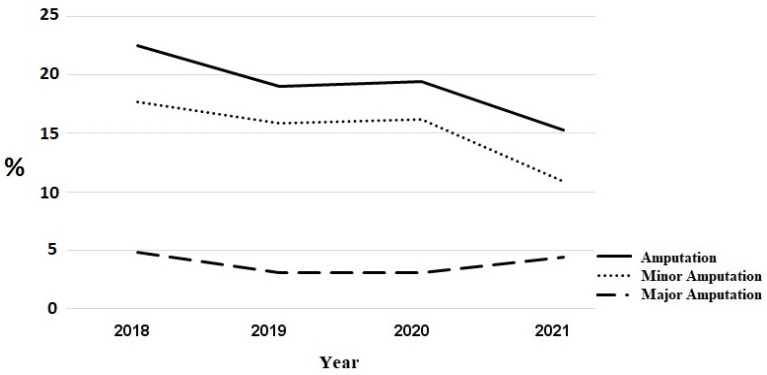
Title: Proportionate course of in-house amputations and transfers for amputation in other hospitals in patients with Rutherford–Becker class 5 (including minor and major). Legend: Proportionate major and minor in-house amputations and transfers for amputation in other hospitals in patients with Rutherford–Becker class 5.

The total number of amputations increased insignificantly from 176 amputations in the pre COVID-19 period to 191 amputations in 2020/2021. Figure 2 shows the course of in-house amputations. However, the proportion of patients transferred to another hospital for amputation decreased. In 2018, 50 patients were transferred (46 patients for minor amputation, 4 patients for major amputation); in 2019 and 2020, 47 patients each were transfered (43 and 45 for minor amputations, 4 and 2, respectively, for major amputations), whereas in 202,1 there were only 38 transfers (6 for major amputations, 32 for minor amputations). The trend in the total number of amputations including in-house amputations and transfers to other hospitals for amputations is shown in Figure 3. During the COVID-19 period, the number of major amputations increased from 43 in the pre-COVID-19 period to 53 amputations, while the number of minor amputations increased from 133 to 138, respectively. However, the proportion of patients with wounds requiring amputation does not differ significantly between the study periods (minor amputations 10% vs. 8.6%, *p* = 0.2302, major amputations 3.2% vs. 3.3%, *p* = 0.980).

## 4. Discussion

Previous studies evaluated the impact of the first wave of the COVID-19 pandemic. The present study investigated the development of in-patient admissions for endovascular treatment of PAD patients and acute clinical outcomes in terms of procedural details and amputation rates during a two-year course of the pandemic, including 2020 and 2021, compared to the pre-COVID-19 era (2018 and 2019).

In this single-center study, no significant decrease in endovascular interventions due to PAD was found during the pandemic period. However, as described in previous studies [6,7], the number of peripheral interventions was at its lowest in 2020. This particular year was marked by a lockdown lasting several weeks and great uncertainty. In order to pool resources, only urgent patient admissions were accepted. The few possible in-patient admissions were reserved for patients presenting mainly with CLTI or acute onset of severe claudication symptoms. Only a few patients with claudication were admitted, as long as those patients had no concerns about the possible risk of acquiring a COVID-19 infection during hospitalization.

The major finding of the present study is the significant increase in endovascular interventions in patients with wounds during the COVID-19 period with no significant increase in acute major and minor amputation rates. The increase in patients admitted with ischemic wounds may be related to a later access to medical care for patients with critically impaired peripheral arterial perfusion related to primary diagnosis and adequate ambulant wound care.

An analysis by an American wound care company showed that patients who received care for newly developed wounds presented with larger and deeper wounds in the COVID-19 year 2020 compared to 2019. While telemedicine care increased, the number of in-person visits per wound decreased [10].

Fortunately, with the increase in interventions of patients with ischemic wounds stage 5 and 6 according to Rutherford–Becker classification, the overall number of acute minor and especially major amputations did not increase significantly. Due to limited transfer capacities to secondary hospitals for amputation, the number of in-house amputations increased.

A study from England showed no increase in the number of minor and major amputations in diabetics during the first COVID-19 wave. Rather, there was a decrease in amputations during this period. However, only data from March to June 2020 were examined. A potential increase in numbers due to delayed referrals cannot be excluded [11].

In contrast, surveys of vascular specialists in the USA report later-stage presentation in patients with CLTI and the performance of more extensive amputations [12,13]. An Italian analysis [14] also showed an increase in amputations in patients with CLTI during the COVID-19 pandemic with an increase in major amputations from 3.3% to 5.4%.

During the COVID-19 period, the complexity of the interventional procedures increased significantly as evidenced by a significant increase in procedural duration and fluoroscopy time. However, the increase in two-level interventions and treatment of infrapopliteal lesions was not significant. Furthermore, the rate of recanalization procedures of total occlusions remained stable. The latter aspect may explain why the amount of contrast medium use was unchanged between the two study periods. On the other hand, the more frequent use of digital subtraction angiography for infra-popliteal interventions—compared to femoropopliteal or iliac interventions, which tended to be more frequent in the COVID-19 period—may have led to the use of higher diluted contrast medium so that the amount of average contrast medium consumption remained stable overall.

Lou et al. evaluated data from the North American quality assurance registry and showed a similar increase in complexity of the endovascular cases during the first pandemic wave. Their study demonstrated an increase in TASC-D lesions, occlusion length, and the proportion of crural interventions [15].

## 5. Limitations

A major limitation of the study is the single-center design including only patients treated in a high-volume center with special expertise in complex interventional procedures. Therefore, the outcomes may not be representative of the average interventional and surgical departments. Secondly, regarding amputation rates, data are only available for the acute in-hospital phase up to the time of discharge. The study is not able to provide mid-term and long-term clinical outcome data.

## 6. Conclusions

The COVID-19 pandemic had an impact on in-patient care for patients with PAD regarding procedural complexity of the endovascular treatment and the presentation with ischemic wounds. Endovascular treatment of these patients in a high-volume center could prevent an increase in acute amputation rates. However, efforts should be made to avoid delaying outpatients’ access to appropriate PAD diagnosis and wound care under pandemic conditions.

## Figures and Tables

**Table 1:** Cardiovascular risk factors.

	2018	2019	2020	2021	*p*-Value ^1^
Mean Age, yrs ± SD	71.8 ± 11	73.3 ± 20.2	72.8 ± 10.4	72.5 ± 10.9	0.010
Male sex *n*,%	1654 (64)	1816 (65)	1610 (63.8)	1754 (65.7)	0.939
Hypertension *n*,%	2329 (90.7)	2479 (88.8)	2254 (89.4)	2405 (90.1)	0.912
Hyperlipidemia *n*,%	2113 (82.3)	2418 (86.6)	2222 (88.1)	2352 (88.2)	<0.001
Diabetes *n*, %	963 (37.5)	1157 (41.4)	1130 (44.8)	1170 (43.9)	<0.001
Current Smoker *n*,%	907 (35.3)	915 (32.8)	799 (31.7)	938 (35.2)	0.588
Former Smoker *n*,%	762 (29.7)	908 (32.5)	803 (31.8)	789 (29.6)	0.611
Dialysis *n*,%	85 (3.3)	91 (3.3)	103 (4.1)	98 (3.7)	0.114

^1^ Comparing both study periods (pre COVID-19 era vs. COVID-19 era).

**Table 2:** Distribution of indications for endovascular intervention comparing the study periods.

Year	Intermittent Claudication *n* (%)	Critical Limb-threatening Ischaemia *n* (%)	*p*-Value
Pre COVID era	3239 (60.4)	2112 (39.6)	<0.001
2018	1572 (61.2)	996 (38.8)	<0.001
2019	1667 (59.7)	1126 (40.3)	<0.001
COVID era	2764 (53.3)	2426 (46.7)	<0.001
2020	1352 (53.6)	1170 (46.4)	0.002
2021	1412 (52.9)	1256 (47.1)	0.013

**Table 3:** Number of interventions according to level involvement.

	2018	2019	Pre COVID-19 Era (18/19)	2020	2021	COVID-19 Era	*p*-Value (Pre vs. COVID-19 Period)
Iliacal	572 (22.3)	583 (20.9)	1155 (21.5)	502 (19.9)	584 (21.9)	1086 (20.9)	0.49
Femoropopliteal	2019 (78.6)	2171 (77.7)	4190 (78.2)	1960 (77.7)	2125 (79.6)	4085 (78.7)	0.69
Infrapopliteal	982 (38.2)	1037 (37.1)	2019 (37.7)	1085 (43.0)	1092 (40.9)	2177 (41.9)	0.10
1-level intervention	1599 (62.3)	1826 (65.4)	3425 (63.9)	1538 (60.9)	1578 (59.1)	3116 (60)	0.31
2-level intervention	918 (35.8)	933 (33.4)	1851 (34.5)	943 (37.4)	1038 (38.9)	1981 (38.2)	0.31
3-level intervention	46 (1.8)	33 (1.2)	79 (1.5)	41 (1.6)	49 (1.8)	90 (1.7)	0.55

**Table 4:** Intervention details (2018/2019 vs. 2020/2021).

	2018/2019 (*n* = 3726)	2020/2021 (*n* = 3677)	*p*-Value
Intervention duration (min)	62.9 ± 31.9	66.0 ± 30.8	<0.001
Contrast volume (mL)	75.5 ± 37.9	75.5 ± 37.1	0.500
Radiation dose (cGy cm^2^)	2144.6 ± 2806.4	2045.0 ± 2852.0	1.00
Fluoroscopy duration (min)	13.9 ± 11.9	14.4 ± 13.3	<0.001

## Data Availability

The data presented in this study are available on request from the corresponding author.
